# Evaluation of Various Inoculation Methods on the Effect of *Beauveria bassiana* on the Plant Growth of Kiwi and on *Halyomorpha halys* Infestation: A Two-Year Field Study

**DOI:** 10.3390/biology13070470

**Published:** 2024-06-26

**Authors:** Vasileios Papantzikos, Spiridon Mantzoukas, Panagiotis A. Eliopoulos, Dimitrios Servis, Stergios Bitivanos, George Patakioutas

**Affiliations:** 1 Department of Agriculture, Arta Campus, University of Ioannina, 45100 Ioannina, Greece; gpatakiu@uoi.gr; 2 Laboratory of Plant Health Management, Department of Agrotechnology, University of Thessaly, Geopolis, 41500 Larissa, Greece; eliopoulos@uth.gr; 3 BASF Hellas S.A., 15125 Marousi, Greece; dimitris.servis@basf.com (D.S.); stergios.bitivanos@basf.com (S.B.)

**Keywords:** *Halyomorpha halys*, *Beauveria bassiana*, *Actinidia deliciosa*, biostimulant, bioinsecticide

## Abstract

**Simple Summary:**

Kiwifruit cultivation since the 1980s has historically played a fundamental financial role, especially for the income of rural areas of Greece, which now is one of the top exporters globally. The incursion of non-indigenous pest species is becoming an issue for the cultivation of kiwi that cannot be easily addressed because the use of chemical insecticides, on the one hand, must be limited, and on the other, they are not always effective against non-target insects. The invasion of the *Halyomorpha halys* pest into some Greek areas recently exhibited these challenges, as it significantly affects kiwifruit production, although there is no targeted way of combating it. In this study, we tried to limit the *Halyomorpha halys* population by using a commercial entomopathogenic fungus (EPF) *Beauveria bassiana* in kiwifruit cultivation; we also studied the biostimulant effect of this strain on plant growth. The entomopathogenic fungus ultimately dually reveals benefits for kiwifruit cultivation: as a bioinsecticide and as a biostimulant. The use of the EPF formulations may bring solutions to deal with some newly introduced entomological pests of kiwi in Greece, with a side benefit of its use being the biostimulant effect.

**Abstract:**

In this study, the bioinsecticidal action of a commercial formulation with *Beauveria bassiana* was evaluated on the new sucking pest in Greece: *Halyomorpha halys*, of the kiwifruit. Additionally, the biostimulant potential of the same formulation was studied on kiwi growth. The application was performed in three different ways in a commercial field of kiwi crop *A. deliciosa* “Hayward” field in Arta, Greece: (i) trunk spray, (ii) root injection, and (iii) trunk inoculation. During the 2 years seasons of the experiment, weekly measurements of the *H. halys* population were determined. The insect is sucking plants nutrients; therefore, the total chlorophyll content in the leaves of the treatments was recorded weekly. In addition, the percentage of infested kiwifruits was estimated at the end of the experiment. Moreover, to study the biostimulant potential of the formulation, growth measurements on stems and leaves were performed during the experiment. Finally, at the kiwi harvest point, the fruit biomass, dimensions, and weight were obtained, and the leaves’ proline content was evaluated. The results encourage us to further study this EPF formulation as the bioinsecticidal effect was noted by the reduction in *H. halys* population, and biostimulant action was perceived by the higher plant biomass.

## 1. Introduction

The commercial crop of “Hayward” kiwifruit (*Actinidia deliciosa*) necessitates high irrigation requirements in order to produce high yield: a situation that more often attracts invasive pests which are usually frequent in crops with high soil moisture [1,2]. During cultivation, infestations by sting bugs are common, and sometimes they are capable of leading to a significantly reduced production, as insects feed on the fruits and leaves with their mouthparts by piercing and sucking out nutrients, causing deformation and tissue damage. The economic value of kiwifruits depends on their robustness, so feeding punctures from sucking pests, in addition to the qualitative loss they cause to the fruits, are also an entry for pathogenic microorganisms that can cause severe damage during the post-harvest life of kiwifruits.

*Halyomorpha halys* (Hemiptera: Pentatomidae) is an exotic invasive pest in Greece, commonly known as the brown marmorated stink bug (BMSB) [3]. It is indigenous to Eastern Asia [4], with a high infestation and spread rate throughout the world [5,6]. This polyphagous pest has a wide host range, including ornamental plants, vegetables, and commercially significant tree crops [5], expressing a preference for crops with frequently irrigated soils such as *Actinidia* ssp. It has been reported as a kiwifruit pest in China [3,7], Korea, Italy, and recently in Greece [3,8], causing significant damage. It has been shown to feed on *A. deliciosa* “Hayward” in parts of Greece [8]. Almost all growth stages of *H. halys* can cause damage to kiwifruits, as both adults and nymphs have been observed feeding on plant leaves or directly on the fruit [8], fruit stems, flowers, fruit buds, or plant shoots [3,9]. Damage occurs when nymphs and adults insert their proboscis [10], piercing the plant surface and secreting a thick salivary compound containing digestive enzymes that aid plant cells in degradation [3,11,12]. The final result is fruit deformation [5] and internal tissue damage [3,8] on the small necrotic areas on the exterior fruit part and on the stalk, which, in most cases, lead to suberifications [10]. The transmission of viral diseases is likely to occur, as the insect carries pathogenic microorganisms [13]. The feeding damage level increases in July, as the population increases and reaches its peak through the summer [4]. Damaged products can significantly lose their commercial value [5] and result in significant financial losses [10]. The wide spread of *H. halys* caused USD 37 million in losses to mid-Atlantic apple growers (American/Western Fruit Grower 2011) [10]. It is expected to spread more in the coming years, as its hosts include a wide range of plant species; for these reasons, it is important to combat. A common farmer’s tactic in dealing with *H. halys* is the use of insecticides [13], which becomes difficult to control, because the insect continuously flies from field to field [4,13]. However, chemical treatment is not consistent with the desired quality of kiwifruit on the market [14]. The effectiveness of several chemical insecticides against *H. halys* in fruit orchard field trials has been investigated, with some of these compounds showing high efficacy in the adult stages of the insect. However, no residual effect has been observed 3–7 days post treatment [15,16]. This fact, together with the negative impacts of insecticides on the environment and human health, shows the need to develop biological control strategies, in order for its application in integrated pest management (IPM) programs, minimizing the excessive use of pesticides. The use of residual pesticides does not present targeted BMSB control; for this reason, there is a need to optimize biological ways of combating the newly introduced pest in Greece.

The use of EPFs (entomopathogenic fungi) provides an additional approach to an IPM program [17], combining a wide range of compatible techniques, such as biological control, to keep fruit infestation below economic damage levels. EPFs act in a wide range of ways, preventing fruit infestations by providing plant metabolism with antioxidant, antibiotic, and antiparasitic properties [18], and producing various secondary metabolites that have unique species-specific bioactive structures (e.g., benzopyranones, phenolic acids, quinones, and steroids) [19]. *Beauveria bassiana* (*Bals.*-*Criv.*) *Vuill.*, (Hypocreales: Cordycipitaceae) is the most widely studied natural EPF endophyte, commonly used as a commercial biological control formulation, and exhibiting a multi-functional lifestyle; it can exist in a variety of ecological environments such as soil, plants, and insects [20]. The effectiveness of *B. bassiana* strains to *H. halys* has been reported in several studies [5,21], as endophytic EPFs [15,22], acting as a beneficial rhizosphere colonizer [23] in a wide range of plants, builds symbiotic relationships [24], benefiting from a multitude of environmental stresses. In addition, it seems to be effective for biological control programs as an environmentally friendly control tool [13]. *B. bassiana* infects insects through the integument, colonizing the hemocoel [25]; then, as it sporulates on the surface of the host, it can cause mortality [5,26]. *B. bassiana* is a chitinase-producing EPF [27], a powerful hydrolytic enzyme important for the development of biopesticide formulations that can successfully control a wide range of invertebrates [28]. Enzyme derivatives of *B. bassiana* not only benefit plants by combating harmful pests, but may also enhance plant metabolism and promote their growth [29]. Therefore, the biostimulant potential of *B. bassiana* has been recorded on many crops [19,30,31,32,33,34,35].

In the present study, a two-year field trial was conducted, testing a commercial strain of *B. bassiana* inoculated in three different ways on *A. deliciosa* trees. After application, the kiwi trees were monitored to study the effects on the population of *H. halys*, as well as the potentiality of the strain as a biostimulant.

## 2. Materials and Methods

### 2.1. Experimental Design

**Figure 1:**
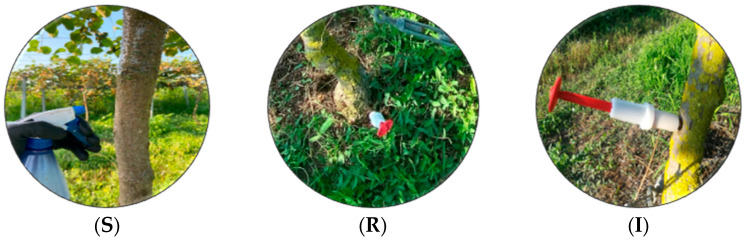
Depiction of *Β. bassiana* application on *A. deliciosa* “Hayward”: (**S**) trunk spray; (**R**) root injection; (**I**) trunk injection.

A plot section of the commercial cultivation of kiwi trees *A. deliciosa* “Hayward” was selected for experimentation, at the geographical location 39.121168262553795, 20.942477410866765, in the region of Arta, Greece. The experiment lasted for 2 seasonal years, from June 2022 to November 2023 (508 days). *Beauveria bassiana* commercial strain PPRI 5339 Velifer^®^ OD (BASF SE, Florham Park, NJ, USA) was used in trials, where four treatments were distributed in a completely randomized design, including the control (C), where only water was applied. Each treatment consisted of three replications, and each replication included twelve trees. We evaluated the efficacy of *B. bassiana*, formulated as Velifer^®^ (10 mL of the original formulation) in 3 different ways: trunk spraying (S), root injection (R), and trunk injection (I). The S formulation was applied by spraying the solution on the trunk of each tree with a sprayer in a radius of 30 cm around the trunk (Figure 1S). Instead of direct rooting, root site injection application, R, was carried out. The formulation was applied on each tree and through a slow-release tree injector syringe (Chemjet, Stratagreen Co., Prestons, WA, Australia) inserted into the soft spot of the trunk at a 45° angle very close to the root (Figure 1R). For I, in each tree of the treatment at a height of 70 cm from its base and with a slope of 45°, a thin bore (4 cm) was drilled with a sterile screw, into which the tree injector containing the solution of the formulation, was inserted (Figure 1I). Within 72 h, the solution was released, passing through the vascular bundles of the tree. Through the osmotic pressure, it was transferred to the upper parts of the plant. When the tree injector was withdrawn from the tree trunk, a grafting paste was applied to the attachment point.

### 2.2. Population of H. halys Measurements

**Figure 2:**
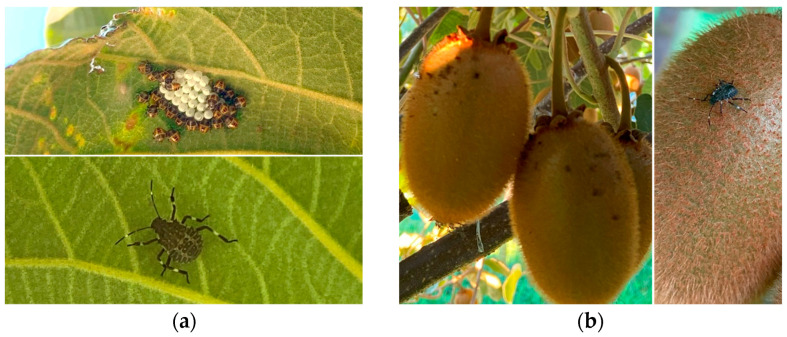
*H. halys* on *A. deliciosa* “Hayward” leaves and fruits of the experiment: (**a**) eggs and 5th instar nymph on leaves; (**b**) fruit infestation represented by necrotic areas on the exterior fruit part.

The population of *H. halys* was systematically recorded on a weekly basis. In order to attract the *H. halys* into the experimental plots, a pheromone capsid was placed on an adhesive surface, in the center of each treatment area. The pheromone capsid was renewed monthly. The total population of *H. halys* (nymphs and adults) on each tree of each treatment was estimated every week. The population and the eggs of *H. halys* were monitored in 20 leaves uniformly distributed in the shaded parts of the crown, at a radius of 50 cm from the branching of the trunk, in each tree, of each treatment (Figure 2a).

### 2.3. Number of Infested Fruits

One hundred kiwifruits per tree were observed randomly from a height of 180 cm above the ground level from four directions (south, east, north, and west), every week. Fruits showing symptoms such as fruit deformation with necrotic areas on the exterior fruit part and on the stalk were considered as infested by the BMSB (Figure 2b). The results were expressed as a percentage of fruit damage.

### 2.4. Plant Growth Parameters

To estimate the biostimulant effect of the *B. bassiana*, plant growth parameters of kiwi trees were measured. On fresh vegetation, shoots of the kiwi trees were measured on-site: the diameter (mm) with a digital slide caliper (Insize Co., Ltd., Suzhou, China ); the length (cm) with a portable ruler; and the number of internodes and leaves. At the end of each season, the following samplings were carried out on-site and stored for a couple of hours, in order to assess them in the lab: (a) the fresh and the dry weight (g) of shoots, leaves, and kiwifruits, after 48 h at 80 ± 1 °C weighed on a precision electronic scale (Kern EG-N, Kern & Sohn GmbH, Balingen, Germany); (b) the dimensions (cm) of kiwifruits, measuring their length and width with a portable meter; and (c) the leaf area (cm^2^), determined by the Image J (v 1.54) protocol according to Bakr, 2005 [36].

### 2.5. Total Chlorophyll Content of A. deliciosa “Hayward” Leaves

To quantify the total chlorophyll content, SPAD values were taken from 40 randomly selected leaves (5 measurement points per leaf) of the productive shoots from all the trees of each treatment. Determination of the total chlorophyll of the kiwi leaves was carried out every week by the non-destructive method provided by the SPAD-502 instrument (Minolta Co., Ltd., Osaka, Japan). SPAD values were linearly correlated with actual chlorophyll units through the conventional chemical determination in some kiwifruit leaf samples (R^2^ = 0.9145), according to the method of Razeto and Valdés [37], with some modifications. Briefly, 10 mL of pure acetone was used as the extraction solvent for 0.04 g of homogenized fresh leaf tissue. Each sample was vortexed and left overnight at 4 °C. The absorbance was determined at 644.8 and 661.6 nm using a spectrophotometer (Jasco-V630 UV-VIS, Jasco International Co., Ltd., Tokyo, Japan), and the chlorophyll of the solutions was determined using the equations published by Lichtenthaler and Buschmann [38], expressed in μg of fresh leaf per cm^2^ of leaf area:

Ca (μg/mL) = 11.24 × A_661.6_ − 2.04 × A_644.8_

Cb (μg/mL) = 20.13 A_644.8_ − 4.19 A_661.6_

### 2.6. Proline Determination

To assess the plant stress, proline determination was conducted. Leaf sampling was conducted monthly, timed to coincide as closely as possible with warmer periods of each season. This approach was intended to monitor the proline values under conditions that permanently induce physiological pressure, as proline is an indicator of abiotic stress. The leaves were cut with sterile scissors from the base of the stem, cleaned with diH_2_O, and immediately placed in sampling bags, which were stored in a portable refrigerator. According to the protocol developed by Carillo and Gibon [39], 0.1 g of fresh kiwi leaf tissue was extracted in 4 mL of 70% ethanol and centrifuged (Heraeus Biofuge, Primo R refrigerated centrifuge, Thermo Fisher Scientific Co., Inc., Waltham, MA, USA) at 4000× *g* for 10 min at 4 °C. In a new glass tube, 2 mL of freshly prepared acid-ninhydrin solution and 1 mL of the supernatant were placed, then vortexed and incubated in a water bath at 95 °C for 25 min. The reaction mixture was cooled directly in an ice bath until room temperature was reached. Then, a new centrifuge for 5 min at 4000× *g* followed, and the absorbance was determined at 520 nm in a spectrophotometer (Jasco-V630 UV-VIS, JASCO International Co., Hachioji, Tokyo, Japan). The results were reported in μmol of proline g^−1^ of fresh kiwi leaf weight.

### 2.7. Statistical Analysis

To compare parameters of the treatments, two-way ANOVA was performed with Tukey’s post hoc test (*p* < 0.05). Two-way ANOVA was performed to evaluate the main effects and interactions of the two main factors: treatment and the time interval (days) after treatment. Statistical analysis was performed using the program SPSS v. 25 (IBM-SPSS Statistics, Armonk, NY, USA).

## 3. Results

### 3.1. Population of H. halys (Adults and Eggs) on A. chinensis Leaves

**Figure 3:**
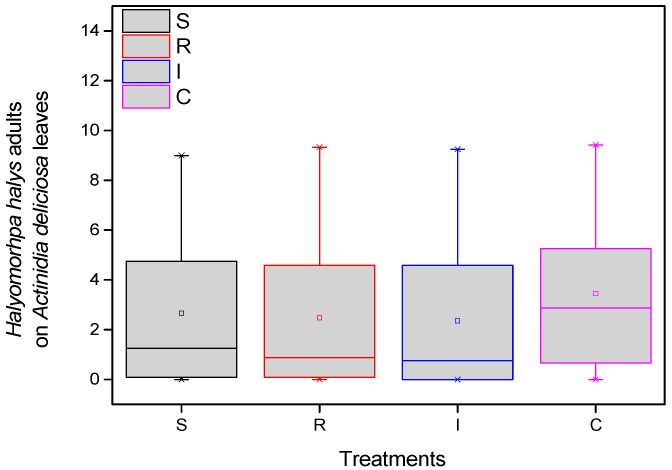
Number of *H. halys* adults per leaf on *A. deliciosa* “Hayward”. One hundred leaves (*n* = 100) were sampled and examined from each treatment plot. Definition of EPF treatments: (S) trunk spraying; (R) root injection; (I) trunk injection; and (C) Control.

The population of *H. halys* adults per treatment in kiwi leaves was recorded. More specifically, in all eight samplings (four in the first year and four in the second year), the differences in the average number of *H. halys* adults among treatments were statistically significant (F = 19.883, df = 3.717, *p* < 0.001), given that significantly more adults were collected from the control compared to treated plants. Fewer adults were almost always counted on plants treated with trunk injection than the other treatments. These differences were significant in most cases (Figure 3).

**Figure 4:**
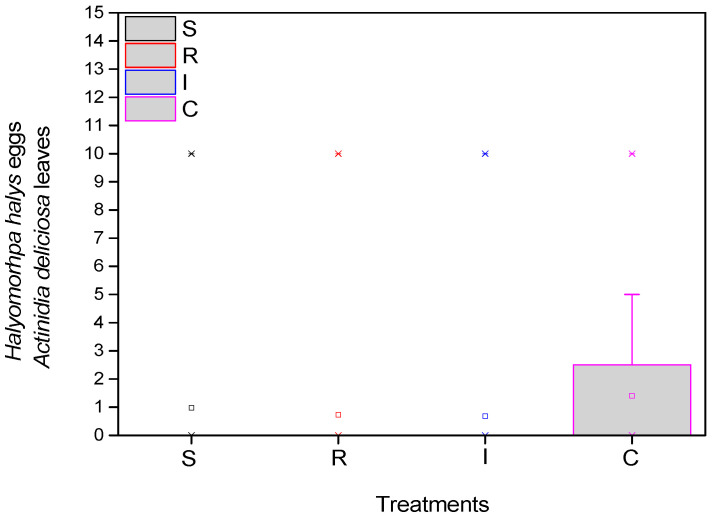
Number of *H. halys* eggs per leaf on *A. deliciosa* “Hayward”. One hundred leaves (*n* = 100) were sampled and examined from each treatment plot. Definition of EPF treatments: (S) trunk spraying; (R) root injection; (I) trunk injection; and (C) control.

As for the eggs of the *H. halys*, the mean number was significantly higher in the control (F = 17.985, df = 3.717, *p* < 0.001), at the beginning of the experiment. There appeared to be statistically fewer average eggs at the end of the experiment in the treatments compared to the control (Figure 4).

### 3.2. Damaged Kiwifruits

**Figure 5:**
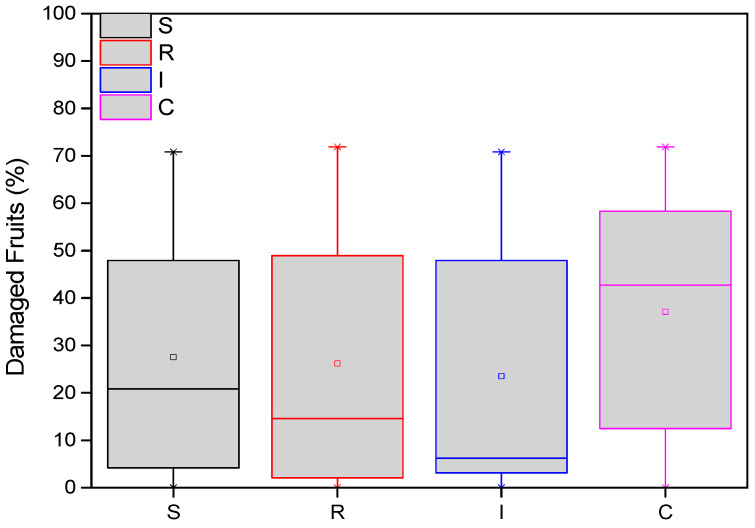
Number of damaged *A. deliciosa* “Hayward” fruits from *H. halys*. One hundred leaves (*n* = 100) were sampled and examined from each treatment plot. Definition of EPF treatments: (S) trunk spraying; (R) root injection; (I) trunk injection; and (C) control.

The average damage of *A. chinensis* fruits was recorded at the end of both years. More specifically, in two samplings (127 days after treatments in the first year and 508 days after treatments in the second year), the variation in the number of damaged fruits from *H. halys* adults was statistically significant among treatments (F = 23.123, df = 3.717, *p* < 0.001). From the end of the first year (November 2022), the average number of damaged fruits was significantly higher in the control than in the treatments. These differences were significant in most cases (Figure 5). Fewer damaged fruits were almost always counted on plants treated with *B. bassiana* trunk injection (I) than the other treatments.

### 3.3. Effect on Plant Growth and Proline Content

Evaluation of the morphological features of the tested plants was based on the recording of the number of leaves, total stem length, stem diameter, number of internodes, and proline content. In general, in the first year, the R- and I-treated plants exhibited more leaves and higher lengths compared to the control. However, differences were not always statistically significant, especially during the second year.

**Figure 6:**
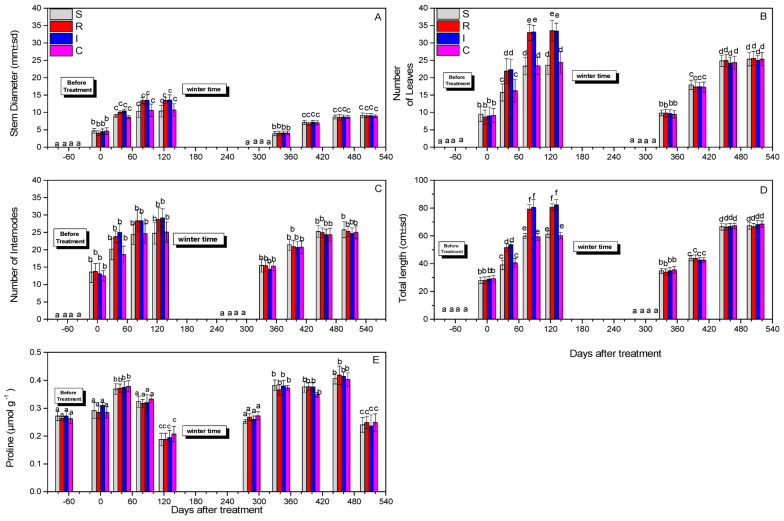
Stem diameter (**A**), number of leaves (**B**), number of internodes (**C**), total length (**D**), and proline content (μmol g^−1^) (**Ε**) of *A. deliciosa* “Hayward” plants inoculated with *B. bassiana* formulation over 508 days after treatment. Different letters between treatments indicate statistically significant differences (Tukey test, *p* < 0.05). Definition of EPF treatments: (S) trunk spraying; (R) root injection; (I) trunk injection; and (C) control.

The stem diameter at both years did not change significantly after 508 days (F = 1.112, df = 3.717, *p* = 0.890) (Figure 6A). The numbers of leaves were not statistically different after 508 days (F = 45.190, df = 3.717, *p* = 0.743) for the treatments compared to the control (Figure 6B). The numbers of internodes after 508 days did not differ significantly (F = 2.111, df = 3.717, *p* = 0.915) (Figure 6C), whereas differences in the total length after 508 days were marginally significant (F = 41.311, df = 3.717, *p* = 0.047) (Figure 6D). Finally, the proline content did not change notably after 508 days (F = 1.814, df = 3.717, *p* = 0.734) (Figure 6E).

### 3.4. Effect on A. chinensis Total Chlorophyll Content (TCHL)

**Figure 7:**
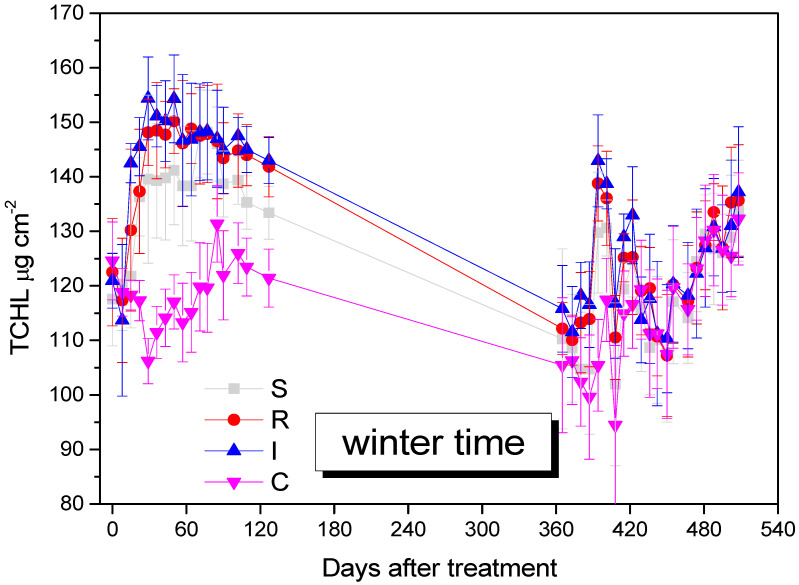
Mean TCHL values of *A. deliciosa* leaves over 508 days post treatment. Different letters between treatments indicate statistically significant differences (Tukey test, *p* < 0.05). Definition of EPF treatments: (S) trunk spraying; (R) root injection; (I) trunk injection; and (C) control.

TCHL was increased in *B. bassiana*-inoculated kiwi trees and remained higher compared to the control kiwi trees until the end of the experiment, after 508 days (F = 29.111, df = 3.577, *p* = 0.009). The increase in TCHL was attributed to the endophytes’ effect, especially for R and I treatments (Figure 7).

### 3.5. Effect on A. chinensis Fruits and Leaf Area

**Figure 8:**
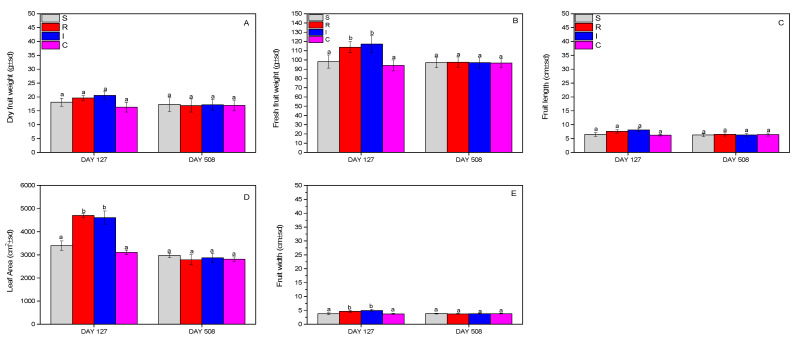
Dry weight of fruits (**A**), fresh weight of fruits (**B**), fruit length (**C**), leaf area (**D**), and fruit width (**Ε**) of *A. deliciosa* “Hayward” plants inoculated with *B. bassiana* formulation over 508 days post treatment. Different letters between treatments indicate statistically significant differences (Tukey test, *p* < 0.05). Definition of EPF treatments: (S) trunk spraying; (R) root injection; (I) trunk injection; and (C) control.

The evaluation of the effect on kiwifruits and leaves was carried out by recording the dry and fresh weight of the fruits, and finally the leaf area. Fruit dry weight and fruit length did not change significantly (dry weight: F = 0.908, df = 3.399, *p* = 0.770; length: F = 2.234, df = 3.215, *p* = 0.445) during the whole experimental period (Figure 8A,C), whereas significant changes were noted for the fresh weight (F = 11.208, df = 3.399, *p* = 0.031) (Figure 8B), leaf area (F = 14.412, df = 3.196, *p* = 0.027) (Figure 8D), and fruit width (F = 19.111, df = 3.399, *p* = 0.023) (Figure 8E), where R- and I-treated plants outperformed the other treatments.

### 3.6. Effect on A. chinensis Biomass

**Figure 9:**
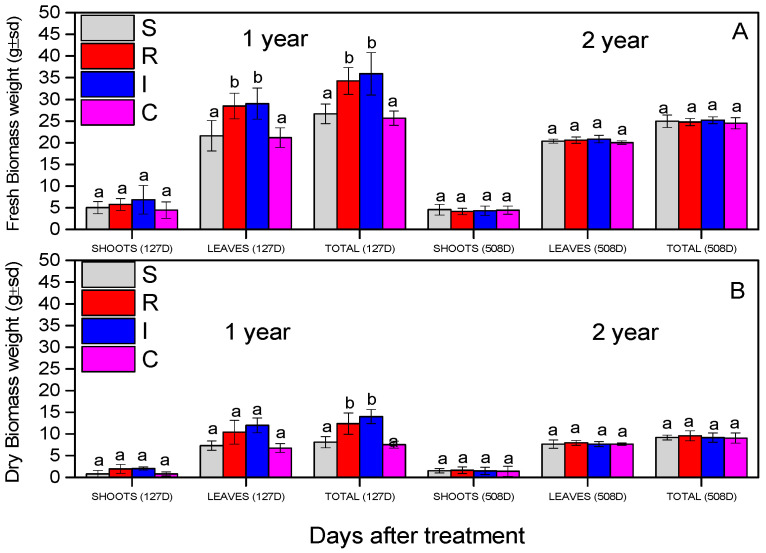
Fresh biomass weight (**A**) and dry biomass weight (**B**) of *A. deliciosa* “Hayward” plants inoculated with *B. bassiana* formulation. Different letters between treatments indicate statistically significant differences (Tukey test, *p* < 0.05). Definition of EPF treatments: (S) trunk spraying; (R) root injection; (I) trunk injection, and (C) control.

The evaluation of the biomass of the *A. chinensis* was based on the recording of fresh and dry biomass (stems and leaves). In all measurements, all the plants were not statistically different during the experiment, except the leaves’ fresh weight and the total fresh and dry weight in the first year, for R and I treatments (Figure 9). The fresh and dry weight of the shoots did not change significantly (fresh shoot weight: F = 1.191, df = 3.110, *p* = 0.693; dry shoot weight: F = 3.345, df = 3.110, *p* = 0.560), as presented in Figure 9A,B. The fresh weights of the leaves (F = 9.719, df = 3.110, *p* = 0.029) and the total fresh weights (F = 19.111), df = 3.599, *p* = 0.011) were statistically significant for the R and I treatments in the first year. In the second year, the leaves’ fresh weight did not differ (F = 2.6459, df = 3.110, *p* = 0.356); finally, the total fresh weights were significantly greater in R- and I-treated plants (F = 17.911), df = 3.599, *p* = 0.023).

## 4. Discussion

The *H. halys* feeding damage on kiwifruit has been referred to in recent years [40,41,42,43,44,45], and may be a potential threat for *A. deliciosa* “Hayward” in Greece. The entomopathogenic effect of *B. bassiana* on invasive pests is a fact, not only found in this study, but also in other research [46,47,48].

There are two reports about the endophytic action of *Beauveria* ssp., on *H. halys* on kiwifruit, both in controlled conditions. The first deals with the evaluation of *B. bassiana* against the nymphs of *H. halys* [13]; the second concerns the efficacy of *B. bassiana* on *H. halys* eggs [49]. These reports are in accordance with our study, especially during the experimental season of 2022. Tozlu et al., 2019 [13] concluded a high mortality rate (76.9%) of *H. halys* nymphs through the application of *B. bassiana*; Mantzoukas et al. [49] reported high toxicity to all *H. halys* stages by *Beauveria varroae*. Decreases in *H. halys* populations by *B. bassiana* have also been observed in hazelnut *Corylus avellana* (Fag6ales: Betulaceae) orchards [5]. In our case study, the spread of *H. halys* was reduced by all three application methods, and much more in the first experimental season. In our study, the trunk inoculation application treatment (I) was much more efficient than all others; this effect was observed for the first time in kiwifruit cultivation. This is probably because endophytic EPFs act more directly when they inhibit tissues that support their faster movement and dispersal to the rest of the plant’s biological organs [50]. Both applications, through injection on the trunk of kiwi trees (I) and on the root (R), are treatment methods from essential plant organs, through which juices and nutrients are moved. According to Hussain et al., 2024 [50], the plant tissues of the trunk are a pathway for the easier movement and dispersion of *B. bassiana* hyphae. Within the tree trunk, *B. bassiana* can move and colonize endophytically in parenchyma and vascular tissue [51], particularly in the petiole [52] in date palm *Phoenix dactylifera* (Acerales: Aceraceae). The upward movement of *B. bassiana* has been confirmed in corn *Zea mays* L., (Poales: Poaceae) tissues [53,54]. This pattern of movement of *B. bassiana* has been observed from the roots to the aerial sections of the plant [55,56]. Whereas, in plants such as *Z. mays*, the majority of research has indicated upward transportation within plant structures, likely occurring in parenchyma and mesophyll tissues, coinciding with photosynthesis, within xylem vessels, and through the air spaces between parenchyma cells [53,54,57]. In our experiment, the distribution of the EPF formulation elements through the vascular bundles of the trunk on the I treatment may be a possible scenario for its successful action as both a bioinsecticide and biostimulant in *A. chinensis*. A similar scenario is likely to occur in the R treatment, which was also tested for the first time on kiwifruit and was observed as effective in reducing the *H. halys* population. The presence of *B. bassiana* in the organs of the roots possibly reduced the *H. halys* population and probably enhanced the kiwi trees’ growth characteristics; however, we believe that this needs further research in order to determine the exact mode of action.

Trunk inoculation by injection (also referred to as macro-injection) is not always a cost-effective approach because it requires well-trained personnel and it is a time-consuming method [58,59,60], although it may have some benefits for specific plant species [61,62]. Other application methods, such as leaf or trunk spraying, may be easier to use. Still, spraying methods sometimes may not demonstrate the same efficiency and residence time in plant tissues, due to various biotic factors, including spore load (CFU mL^−1^) and concentration, dispersal and spore persistence, shelf-life, and virulence [63,64]. Nevertheless, there are cases of crops in which there was no beneficial effect noted from the endophyte application on them with trunk penetration techniques, such as in avocado *Persea americana* (Laurales: Lauraceae) [58,59,60]; some methods may be species-specific [61].

The spray application (S) showed lower efficacy compared to the other methods (I, R), probably because the endophytes are affecting stronger when inhabiting the internal tissues of the plant [65,66]. Moreover, the successful endophytic action and colonization of *B. bassiana* in the root area has also been observed on other commercial crops [61,67]. Of course, the foliar mode of action in other experiments has shown beneficial effects in other plants such as tomato *Solanum Lycopersicum* L. (Solanales: Solanaceae) [68] and sorghum *Sorghum bicolor* (Poales: Poaceae) [69], but this was not observed to a significant extent in our experiment.

The application of *B. bassiana* on crop growth is effective on the overall vegetative growth of various plants such as *S. lycopersicum* [32] and rice *Oryza sativa* L. (Poales: Poaceae) by increasing leaf area, photosynthetic pigments, and proline levels [70], and on leaves of oilseed rape *Brassica napus* (Brassicales: Brassicaceae), inducing the biosynthesis of several flavonoids [71]. In addition, *B. bassiana* has shown biostimulant properties on grapevine *Vitis vinifera* L., (Vitales: Vitaceae) [72] by increasing root growth. EPFs have been shown to have plant-growth-promoting effects through various mechanisms of assisting the growth of plant metabolism in a multitude of studies [73,74]. Some EPFs are plant growth regulators, enhancing the production of auxin and gibberillic acid (GA) and indole-3-acetic acid (IAA) hormones [74,75,76,77], which induce shoot and root development, and contribute to cell division and elongation, as well as vascular tissue differentiation [78]. Moreover, EPFs provide access to plant nutrients. For instance, the nutrient acquisition mechanism employed by *B. bassiana* on beans *Phaseolus vulgaris* (Fabales: Fabaceae), includes the release of nutrients from insects that are decayed by microbes [74,76,79].

In our study, the increase in total chlorophyll in kiwi leaves was consistent, on the one hand, with the application of the formulation with *B. Bassiana*, which enriches the plant metabolism, and on the other hand with the decrease in *H. halys* population that the formulation causes. This insect sucks; therefore, its presence reduces the chlorophyll content by consuming it, along with the rest of the plant tissue juices. The reduction in the *H. halys* population by *B. bassiana* is possibly associated with the retention of a greater percentage of chlorophyll in plant tissues. At the same time, the existence of *B. Bassiana* in the plant tissues of the kiwi may double the amount of chlorophyll, because it benefits the metabolic processes of the plant tissues, as has been mentioned in other studies. These data in our study are in agreement with the study by Geroh et al., 2014 [80], where, in okra plots treated with *B. bassiana*, an increase in the chlorophyll of the leaves was observed for two reasons: firstly, due to the pathogenic effect of *B. bassiana* on *Tetranychus urticae* (Trombidioformes: Tetranychidae); secondly, due to its enhancing effect in plant metabolism [80]. A corresponding increased amount of chlorophyll has been observed in *Eucalyptus* ssp. (Myrtales: Myrtaceae) seedlings inoculated with *B. Bassiana* to prevent damage after infestation by galling wasp *Leptocybe invasa* (Hymenoptera: Eulophidae) [81]. In experiments by Akter et al., 2023 [70], where *B. Bassiana* was applied to rice, increases in total chlorophyll were observed in both normal and salt stress conditions. Corresponding increases in chlorophyll have also been observed in application experiments on lettuce *Lactuca sativa* L., (Asterales: Asteraceae) [82], clove *Syzygium aromaticum* (Myrtales: Myrtaceae) [83], barley *Hordeum vulgare* (Poales: Poaceae) [84], and tobacco *Nicotiana benthamiana* (Solanales: Solanaceae) [67]. *B. bassiana* demonstrates biostimulant relevance in kiwifruit cultivation, and the I and R application methods, could have been chlorophyll-enhancing factors in our experiment.

Growth characteristics of kiwifruit trees, such as total length leaf area, number of leaves, and fresh and dry weight, were strengthened in our study, especially in the first year, which may be an indication of the potential biostimulant effect of *B. bassiana*. In experiments with *B. Bassiana* application to tomato, increases in dry biomass and the total length of the plant vegetation were observed [85], and when applied to wheat *Triticum aestivum* (Poales: Poaceae), it triggered induced systemic resistance and was beneficial for plant growth [86]. In a study of *B. bassiana* application by inserting hyphae through a small wound in the stem of corn, the total plant and root lengths were increased, and the total chlorophyll content was higher [87], a fact that also agrees with our study. Proline, as an indicator of abiotic stress in plant metabolism [88], presented the same physiological levels in all treatments, which revealed the absence of abiotic stress in all treatments in the presence of the formulation.

The action of the formulation was also beneficial for kiwifruits. The kiwifruit’s total weights and dimensions in the experimental season of 2022 were greater in treatments with *B. bassiana*. Of course, the increased growth of kiwifruits in treatments with *B. bassiana* in our experiment may also have been linked to the stronger absence of the *H. halys* population in them, due to the entomopathogenic effect of *B. bassiana*. *H. halys* wounds the fruits to suck out its juices, and thus alters them qualitatively, which had a detrimental impact on the weight of the kiwifruits due to juice removal or due to the invasion of phytopathogenic fungi. Therefore, the presence of *B. bassiana* possibly improved the kiwifruits, with this result being evident in the lower number of fruit infestations in treatments I and R. These data seem to be positive, but for their further validity, more field experiments should be performed for more years in kiwi crops.

## 5. Conclusions

We studied the extreme scenario of a single application of the *B. bassiana* formulated strain PPRI 5339 in three different ways on kiwifruit plots, at the beginning of the first experimental year. Our aim was to monitor the persistence limits of the EPF formulation throughout the first experimental season and its possible duration during the second, through the recording of growth and metabolic plant parameters. Firstly, our purpose was to investigate the survival ability of the strain, and secondly, using the least possible repetitions (dosages), the aim was to prevent the overuse of the biological preparation, which benefits farmers as a more affordable and single-treatment option. In the second year of the experiment, the viability of *B. bassiana* did not appear as strong as observed through the study of the *H. halys* population and *A. chinensis* growth parameters. This is likely related to the inability of the EPF formulation to survive strongly after one year of application. However, the positive results, both in the reduction in the *H. halys* population and in the enhancement of the growth and metabolic characteristics of the kiwi crop, only became visible in the first experimental season. The aforementioned data lead us to the conclusion that a single trunk inoculation of the formulation at the beginning of each growing season is more likely to maintain this positive effect on kiwi cultivation, both as a bioinsecticidal on the invasive pest *H. halys* and as a biostimulant on *A. chinensis* growth. Therefore, we consider it necessary to carry out a larger volume of studies like ours in order to confirm these data as attributes of this EPF formulation, given the risk posed by the feeding preference of *H. halys* on *A. deliciosa* “Hayward” [89].

This study showed, for the first time, that in the treatments where the Velifer formulation was applied only once with the entomopathogenic fungus *B. bassiana* strain PPRI 5339, the population of the new insect pest of the kiwifruit *H. halys* was greatly reduced. In addition, the percentage of fruit infestations decreased to a very significant extent, while the total chlorophyll increased. This is probably due to the fact that the treatments S, R, and I did not undergo serious sucking damage by *H. halys*, because of the treatment. Finally, among the S (trunk spraying), R (root injection), and I (trunk injection) treatments, the most efficient in almost all parameters was found to be the last one (I).

## Data Availability

The data presented in this study are available on request from the corresponding authors, V.P. and S.M.
